# A Concerted Action of Engrailed and Gooseberry-Neuro in Neuroblast 6-4 Is Triggering the Formation of Embryonic Posterior Commissure Bundles

**DOI:** 10.1371/journal.pone.0002197

**Published:** 2008-05-21

**Authors:** Sophie Colomb, Willy Joly, Nathalie Bonneaud, Florence Maschat

**Affiliations:** 1 Human Genetics Institute, Montpellier, France; The Rockefeller University, United States of America

## Abstract

One challenging question in neurogenesis concerns the identification of cues that trigger axonal growth and pathfinding to form stereotypic neuronal networks during the construction of a nervous system. Here, we show that in Drosophila, Engrailed (EN) and Gooseberry-Neuro (GsbN) act together as cofactors to build the posterior commissures (PCs), which shapes the ventral nerve cord. Indeed, we show that these two proteins are acting together in axon growth and midline crossing, and that this concerted action occurs at early development, in neuroblasts. More precisely, we identified that their expressions in NB 6-4 are necessary and sufficient to trigger the formation of the PCs, demonstrating that segmentation genes such as EN and GsbN play a crucial role in the determination of NB 6-4 in a way that will later influence growth and guidance of all the axons that form the PCs. We also demonstrate a more specific function of GsbN in differentiated neurons, leading to fasciculations between axons, which might be required to obtain PC mature axon bundles.

## Introduction

The Central Nervous System (CNS) assembles a large number of neurons in a stereotypic network. Understanding how these connections are established during development in order to form a properly functioning nervous system is a fundamental question in biology. Much of this wiring takes place during embryonic development. Transcription factors that are highly conserved from Drosophila to humans have been found to be required for specific axon guidance events [1], [2], [3], [4].

In Drosophila, formation of the CNS starts with the delamination from the neuroectoderm of about 30 neuroblasts (NBs) per hemisegment. These NBs delaminate in five different waves (S1 to S5) that occur from stage 8 to stage 11 [5]. Each NB acquires a unique identity according to its position along the dorso-ventral and antero-posterior axes as well as to the timing of its birth; these unique identities are established by virtue of the expression of different transcription factors [6].

A number of Drosophila segmentation genes, which are highly conserved in vertebrates, are responsible for generating both the epidermal and neural patterns within each segment [7] [8]. One such gene, Engrailed (EN), which encodes a homeodomain transcription factor, has been shown to have such a dual function [9]. In addition, the *gooseberry* locus (*gsb*), whose vertebrate homolog belongs to the *Pax* gene family, has been shown to play a critical role in specifying NB fate. The *gsb* locus contains two highly homologous transcripts, *gsb* (or *gsb distal*) and *gsb-neuro* (*gsbN* or *gsb proximal*). *gsbN* is expressed in the descendants of Gsb-positive NBs and thus probably provides continued *gsb* function in these cells [7]. In early neurogenesis, these segment polarity genes are involved in both the formation of NBs and in the specification of their identities [6].

In the Drosophila CNS, embryonic NBs undergo multiple asymmetric divisions whereby they self-renew and produce intermediate progenitor cells, called Ganglion Mother Cells (GMCs). GMCs divide only once, giving rise to two post-mitotic cells that differentiate into neurons and glial cells. Accordingly, each neuroblast produces a nearly invariant number of neuronal and glial cells [10]. Once the NBs are specified, their further development is largely controlled by their intrinsic properties, which are likely determined by the distinct combination of genes expressed in NBs [11] [12].

Once neurons are formed, a subsequent critical phase of early development is the establishment of specific connections between neurons and their target cells. The leading edge of an axon, termed the “growth cone,” navigates over significant distances with great precision. Growth cones guide axons by functioning as exquisite sensors that detect and subsequently respond to a variety of environmental cues [13]. These cues can exist as diffusible or cell surface-associated forms that regulate pathfinding, in which Netrin/DCC and Slit/Robo play a crucial role [14]. Cell surface receptors residing on growth cones and their associated axons interpret these signals as positive/attractive or negative/repulsive forces that act to shape the trajectory of a given pathfinding axon.

The first neurons to extend their axons, named “pioneers” [15], must navigate in an environment devoid of other axons. Subsequently, axons from later differentiating neurons, the so-called “follower” neurons, contact the axons of the pioneers and fasciculate with them to form the mature axon bundles that form the Ventral Nerve Cord (VNC). Axons in the VNC are organized in a simple ladder-like pattern. Indeed, axons either cross the midline to form the anterior and posterior commissures (ACs and PCs, respectively) or form the longitudinal tracts.

This makes the identification of the different cues necessary for axonal growth and pathfinding particularly challenging to understand how these different bundles are constructed according to stereotypic neuronal networks.

Regulatory interactions between *en/inv* and *gsb* (not *gsbN*), have been previously described during neurogenesis, in particular concerning a close relationship in the formation and the specification of the NBs, starting at early stages during the S1 phase of NB delamination [9] [16]. Later, at stage 10, i.e. when the S3 wave of neuroblast delamination takes place, *gsb* is activating *gsbN* [17].

In this report we identified a concerted action of EN and GsbN, starting during the S3 phase, that is crucial to form the PCs, and that occurs independently of the formation of the NBs. Indeed, we show here that expressions of Engrailed and Gooseberry-Neuro transcription factors are crucial to trigger the formation of the PC posterior commissures, whereas a later function of Gooseberry-Neuro leads to fasciculations between axons to form the PC bundles.

Using a two-hybrid screen in yeast, we first identified Gooseberry-neuro (GsbN) as an interacting partner of Engrailed (EN); this result has been confirmed both *in vitro* and *in vivo*. We found that EN and GsbN act together during neurogenesis to form PCs, which shapes the embryonic VNC. Interestingly, we found that this concerted action occurs at early stage in the neuroblasts. Common expression of EN and GsbN in a few NBs of rows 6 and 7 suggested that at least one of these NBs might be involved in the formation of the PCs. Indeed, using a series of rescue experiments, we determined that the expression of EN and GsbN in NB 6-4 is crucial to trigger growth and crossing of the midline of axons that form the PC bundles. We also identified that EN and GsbN proteins might also act independently at later stages, when neurons are differentiated. In particular, we found that axons from GsbN-expressing neurons show fasciculations, suggesting that GsbN might be a crucial factor for axonal guidance of followers.

## Results

### Gooseberry-Neuro interacts with Engrailed

**Figure 1:**
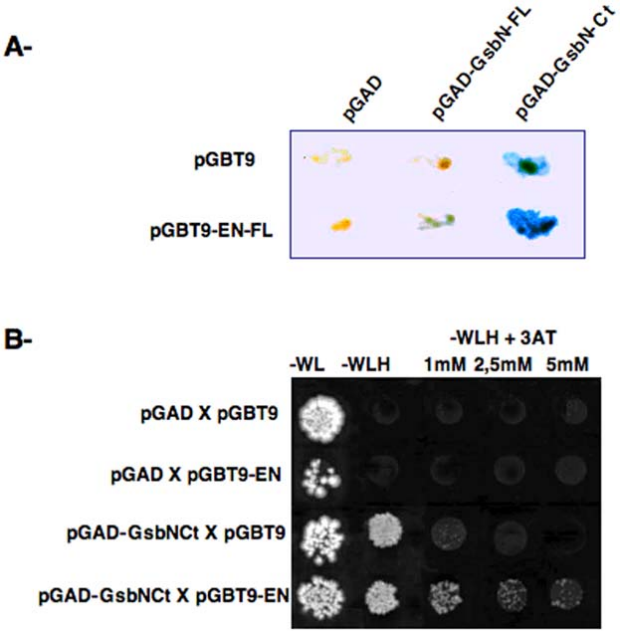
Two-hybrid assay in yeast between EN and GsbN. Assays were performed by mating a *Mata* yeast strain (containing the EN bait) to *Matα* strains (containing GsbN proteins). A ß-Galactosidase staining after mating of a yeast strain containing a sequence encoding the full-length EN protein (EN-FL), cloned in the pGBT9 vector, with strains encoding either full-length (GsbN-FL) or C-terminal (GsbN-Cter) GsbN proteins, cloned in the pGAD vector. B Histidine selection performed on –W-L-H medium, supplemented with increasing amounts of 3′-amino triazol (3AT), as indicated. Empty pGBT9 and pGAD vectors were used as negative controls. Only strains carrying both EN-FL and GsbN-Cter were able to grow in the presence of 3AT.

In an attempt to gain insight into the function of the Drosophila Engrailed (EN) transcription factor, we performed a two-hybrid screen in yeast to search for EN-interacting proteins. We used a construct containing the entire EN protein as bait to screen an embryonic cDNA library. From this screen, around 1000 clones were obtained through Histidine selection, from which we obtained 25 ß-Galactosidase positive clones. These clones were further processed for PCR amplification and sequencing. Among these clones, we identified the GsbN protein as a potential EN-interacting protein. Using a ß-Galactosidase assay, we determined that both the full-length and the C-terminal region of GsbN can interact with EN (Figure 1A). The C-terminal region of GsbN contains the homeodomain, but not the Paired domain, and show low similarities with the Gsb protein, suggesting that this interaction is likely to be specific of GsbN, but not Gsb. On the other hand, interactions were only detected with the full-length EN protein (Figure 1A and data not shown).

Using Histidine selection with increasing amounts of 3AT, we were able to confirm a specific interaction between full-length EN and the C-terminal region of GsbN (Figure 1B).

**Figure 2:**
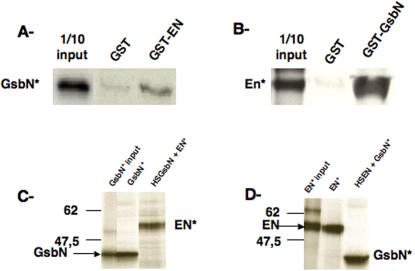
Specific i*n vitro* interactions between EN and GsbN proteins. A and B: GST-pull down assays, A of S^35^-radiolabelled full-length GsbN protein with GST (negative control) or GST-EN fusion protein, and B of S^35^-radiolabelled full-length EN protein with GST (negative control) or GST-GsbN fusion protein. In each case, control of migration corresponds to 1/10 of the input. C and D: Co-immunoprecipitation between EN and GsbN. C Immunoprecipitation of S^35^-radiolabelled GsbN was first tested (GsbN* IP), showing that guinea pig anti-GsbN was able to retain GsbN protein. Co-immunoprecipitation was performed using S^35^-radiolabelled EN protein in the presence of protein extracts from HS-GsbN embryos. D Immunoprecipitation of S^35^-radiolabelled EN was first tested (EN* IP), showing that 4F11 anti-EN was able to retain EN protein. Co-immunoprecipitation was performed using S^35^-radiolabelled GsbN protein in the presence of protein extracts from HS-EN embryos.

To further test the specificity of this interaction, we performed different *in vitro* assays (Figure 2). GST pull-down assays were performed using either ^35^S-labelled GsbN protein (GsbN*) (Figure 2A) or ^35^S-labelled EN protein (EN*) (Figure 2B). These assays showed that the GsbN* protein specifically interacts with GST-EN, while it was barely retained on GST beads alone (Figure 2A). Similarly, EN* protein specifically interacted with GST-GsbN (Figure 2B).

We also performed co-immunoprecipitation (coIP) assays. For this purpose, we first raised a specific antibody against GsbN in rabbit that does not cross-react with Gsb (data not shown). As shown in Figure 2C, when incubated with embryonic extracts expressing high levels of GsbN protein, GsbN was specifically retained on a resin to which anti-GsbN was bound. ^35^S-labelled *in vitro* translated EN protein (EN*) was co-immunoprecipitated with GsbN retained by the anti-GsbN antibody. Inversely, ^35^S-labelled *in vitro* translated GsbN protein (GsbN*) was co-immunoprecipitated with EN using an anti-EN antibody incubated with embryonic extracts expressing high levels of EN protein (Figure 2D). These experiments showed that EN and GsbN proteins are able to specifically interact, at least *in vitro.*

### Engrailed and Gooseberry-Neuro act as cofactors on common target genes

These results suggested that EN and GsbN might interact *in vivo* and act as cofactors in the regulation of common target genes. Immunostaining of polytene chromosomes has been used extensively to map direct EN binding sites, and has led to the identification of several direct targets of EN regulation which have been analyzed and confirmed [18], [19], [20]. A complementary approach, using *in vivo* chromatin immunoprecipitation, has provided a nice picture of direct embryonic EN targets at a genomic scale [21].

In order to explore the possibility that EN and GsbN act together on common target genes, we first analyzed whether they shared common binding sites on polytene chromosomes.

**Figure 3:**
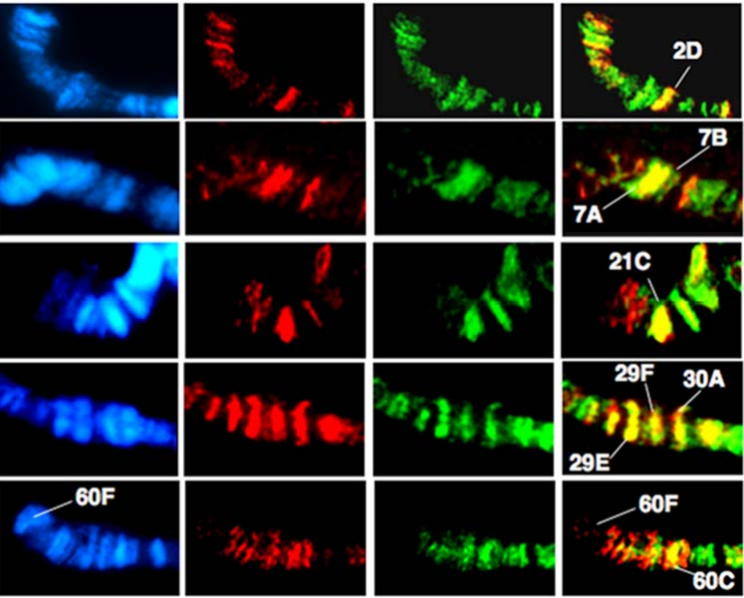
Analysis of EN and GsbN binding sites on polytene chromosomes. Salivary glands from MS1096-Gal4/, UAS-GsbN; UAS-EN L3 larvae were squashed and immunostained with both Rabbit polyclonal anti-EN antibody (detected in red) and guinea-pig polyclonal anti-GsbN antibody (detected in green). DAPI staining was used to visualize the chromosomes. Merged images of the stainings allowed the chromosomal locus identification of common binding sites shown in yellow. Note that chromosomes are presented with the telomere on the left.

For this purpose, we used the UAS/Gal4 system [22] to target expression of both EN and GsbN in larval salivary glands. Squashes of third instar larval salivary glands from the MS1096-Gal4/UAS-EN; UAS-GsbN strain were immunostained with both anti-EN (visualized in red) and anti-GsbN (visualized in green) antibodies (Figure 3). These experiments allowed the identification of 20 to 30 common binding sites. Eight of them, which appear in yellow in Figure 3, were mapped according to banding patterns as visualized by DAPI staining.

Some of the common EN/GsbN binding sites visualized in Figure 3 correspond to regions that have been previously mapped as EN binding sites, but for which target genes have not yet been identified. For instance, regions 7A and 7B have been reproducibly identified as strong EN binding sites (unpublished results) and are shown here to also bind GsbN. Similarly, region 29F, which contains the SoxN locus, an important factor in neurogenesis [23], has been reproducibly found to bind EN (unpublished results) and also binds GsbN (Figure 3). We were also able to identify a common EN/GsbN binding site in the 21C region, where EN-binding genomic fragments have been isolated in chromatin immunoprecipitation experiments [21]. Finally, we were able to identify common EN/GsbN binding sites corresponding to loci in which EN-regulated genes have been previously identified [18], [19], [21]. For instance, the 2D region, which contains *polyhomeotic*, a well characterized direct target of EN that is highly expressed in the CNS [18], was found to bind both EN and GsbN (Figure 3). Likewise, GsbN bound the 60C region, which contains the *ß3-tubulin* gene (Figure 3) which was previously identified as a direct target of EN regulation [19]. This part of the genome, corresponding to the tip of the 2R chromosome, also contains the *gsb/gsbN* locus in the 60F region. As shown in Figure 3, EN did not bind the 60F locus, confirming previous studies [19] and suggesting that GsbN itself is unlikely to be a direct target of EN regulation.

These results confirmed the *in vitro* EN/GsbN interaction and suggested that EN and GsbN might also interact *in vivo* as cofactors to regulate common target genes.

### Engrailed and Gooseberry-Neuro are coexpressed in the VNC

If EN and GsbN act together as cofactors during neurogenesis, we would expect to find neural cells that coexpress both EN and GsbN. The *en/inv* locus, which uncovers EN and its sister Invected (INV) [24], and the *gsb/gsbN* locus [17], has been described as being expressed in a subset of neuronal cells. Coexpression of EN/INV and Gsb/GsbN has also been reported [17].

**Figure 4:**
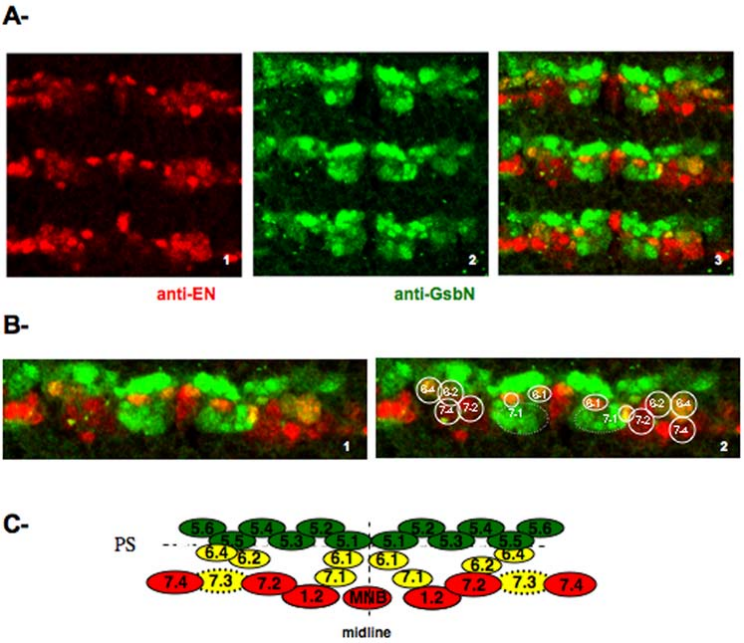
Analysis of EN and GsbN expression in the embryonic VNC. A Stage 11 embryos were immunostained with (1) the 4F11 monoclonal anti-EN antibody, detected in red, and (2) the guinea-pig polyclonal anti-GsbN antibody, detected in green, and immunostainings were visualized by confocal microscopy. (3) Merged images show overlapping expression of EN and GsbN in yellow. B Enlargements of one segment (1). With the annotated position of NBs and/or their progeny (2). Note that NB 7-1 is deliminating from the S1 wave, and all the cells express GsbN, but only one cell express both EN and GsbN. At that stage NB 7-3 did not delaminate yet, but was shown otherwise to express both EN and GsbN (data not shown). C Schematic representation of CNS precursors with EN and GsbN expressions, according to Broadus et al. (1995) [25].

Since we have been interested in functions depending specifically on both EN and GsbN, we verified the presence of cells that express both transcription factors in the VNC. For this purpose, we first raised a specific antibody against GsbN that do not cross react with Gsb (data not shown) and performed double fluorescent immunostainings followed by confocal microscopy (Figure 4). Since GsbN appears only when the S3 wave of NBs takes place, we performed double immunostainings of early stage 11 embryos using a specific anti-GsbN antibody made in guinea pig (in green) and a mouse monoclonal anti-EN antibody (in red). As shown in Figure 4A, we confirmed that at stage 11, GsbN was expressed in rows 5 and 6 and in a few NBs/neurons of row 7. Since EN is also expressed in rows 6 and 7, we were able to identify NBs and/or their progeny that expressed both EN and GsbN (labeled yellow; Figure 4A3). According to the schematic representation of CNS precursors proposed by Broadus et al. [25] (Figure 4C), we identified on enlargement of one segment (Figure 4B), that the overlapping expression of EN and GsbN likely corresponds to NBs 6-1; 6-2; 6-4; 7-1 and/or to their progeny. According to Bossing et al. (1996) [26], NB 7-1 that forms during S1 is localized in the posterior region of *gsb* expression domain. We identified that only one cell in the cluster coexpress both EN and GsbN (Figure 4B). Note also that NB 7-3 only delaminates at S5 and is not yet formed in early stage 11 embryos presented on Figure 4A. However we identified expression of EN and GsbN in NB 7-3 progeny at later stages (data not shown).

Therefore, based on previous work [5], [26], [27] [28] and this study (Figure 4A and Figure 4B), the overlapping expression of EN and GsbN might correspond to NBs 6-1; 6-2; 6-4; 7-1 (and later 7-3) and/or to their progeny.

### Concerted action of Engrailed and Gooseberry-Neuro in the formation of posterior commissures

**Figure 5:**
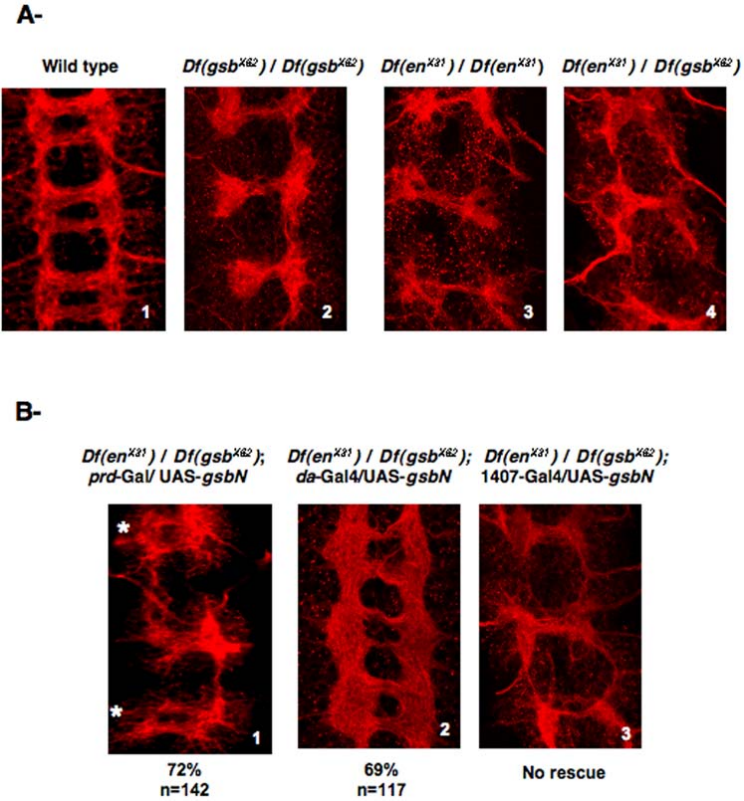
Involvement of EN and GsbN in the formation of the PC commissures. Flat preparations of stage 15 embryos labeled with a Cy3-conjugated anti-HRP antibody to visualize the VNC architecture (red). Dissected embryos are oriented anterior up, and pictures correspond to stacked confocal pictures. A Embryonic VNC architecture of: (1) Wild-type embryos, (2) Homozygous Gsb/GsbN mutant embryos, (3) Homozygous EN/INV mutant embryos, and (4) Transheterozygous EN/INV and Gsb/GsbN mutant embryos. B- Involvement of GsbN expression tested by rescue experiments in transheterozygous embryos shown in A4: (1) with *paired*-Gal4 driver (* show the even segments), (2) with early ubiquitous *daughterless*-Gal4 driver, and (3) with late neuronal *1407*-Gal4 driver. Penetrance of the rescue is indicated, n corresponding to the number of segments.

Homozygous *Gooseberry* mutants were previously described as presenting commissural axon defects, with reduced or missing posterior commissures in each segment [8]. Using a deficiency (*Df gsb^X62^*) that uncovers both *gsb* and *gsbN*, we analyzed the VNC architecture by anti-HRP immunostaining and confirmed that the PCs were not properly formed (Figure 5A2) and were often missing when compared to wild-type (Figure 5A1). Also, EN has been previously shown to be involved in the formation of the PC [12], and analyzing the architecture of the VNC in *en/inv* homozygous mutants (*Df en^X31^*) revealed that the VNC was missing PCs, similar to the *gsb/gsbN* mutant phenotype (Figure 5A3). When tested as heterozygotes individually, both mutants (*Df en^X31^/+*) and (*Df gsb^X62^*/+) showed normal VNCs (data not shown). To address whether EN and GsbN act together in the formation of the PC, we analyzed the VNCs of transheterozygous (*Df en^X31^/Df gsb^X62^*) embryos. In these embryos, we found that 69% of the embryos analyzed (n = 145) presented posterior commissures that were affected (Figure 5A4). Among them, 88% of the segments were presenting a missing PC phenotype (n = 101).

We knew from previous work that both EN and, to a lower extent, INV are involved in the formation of PCs [12]. In addition, Gsb has previously been shown to be important in this process [8]. Since we are specifically interested in interactions between EN and GsbN, rather than Gsb, we first determined whether GsbN is also involved in the formation of the PCs. To answer this question, we performed rescue experiments in the transheterozygous (*Df en^X31^/Df gsb^X62^*) genetic background. For this purpose, the UAS/Gal4 system was used in order to target GsbN expression to specific cells and to analyze its effect on VNC architecture. We used the *paired*-Gal4 driver to express *gsbN* in even, but not odd, segments, thus providing an internal control [29], [12]. In these conditions, rescuing GsbN expression in even segments was able to rescue the missing PC phenotype observed in (*Df en^X31^/Df gsb^X62^*) embryos in 72% of the even segments (n = 142) (Figure 5B1); the odd segments, however, still showed abnormal PCs. This showed that GsbN is involved, together with EN, in the formation of PCs.

To determine when GsbN expression is involved in the formation of PCs, we used various Gal4 drivers to express GsbN in (*Df en^X31^/Df gsb^X62^*) embryos at different times during development. We first expressed GsbN using a *daughterless*-Gal4 driver, which allowed ubiquitous GsbN expression starting in very early developmental stages, meaning that GsbN was already overexpressed when NBs were forming [30]. This was able to rescue the architecture of the nerve cord in (*Df en^X31^/Df gsb^X62^*) embryos in 69% of the segments (n = 117) (Figure 5B2). On the contrary, expression of GsbN in already differentiated GMCs/neurons using a late driver such as 1407-Gal4 [31] did not allow any rescue (n>100) (Figure 5B3).

These results suggested that EN and GsbN are involved together in the formation of the posterior PC commissures, likely through a concerted action. As previously shown for EN [12], GsbN is also not required in this process in differentiated neurons, suggesting that the presence of both proteins is probably necessary in the neuroblasts to control their further development.

### Identification of neuroblasts involved in the formation of the PCs

The observation that PCs are generally missing in transheterozygous (*Df en^X31^/Df gsb^X62^*) embryos (Figure 5A4) suggested a concerted action of EN and GsbN to form these commissures. In addition, as showed above, their expression in this process is required already at early stages, previously to neuronal differentiation (Figure 5B), suggesting that NBs expressing both genes might trigger the formation of PCs.

**Figure 6:**
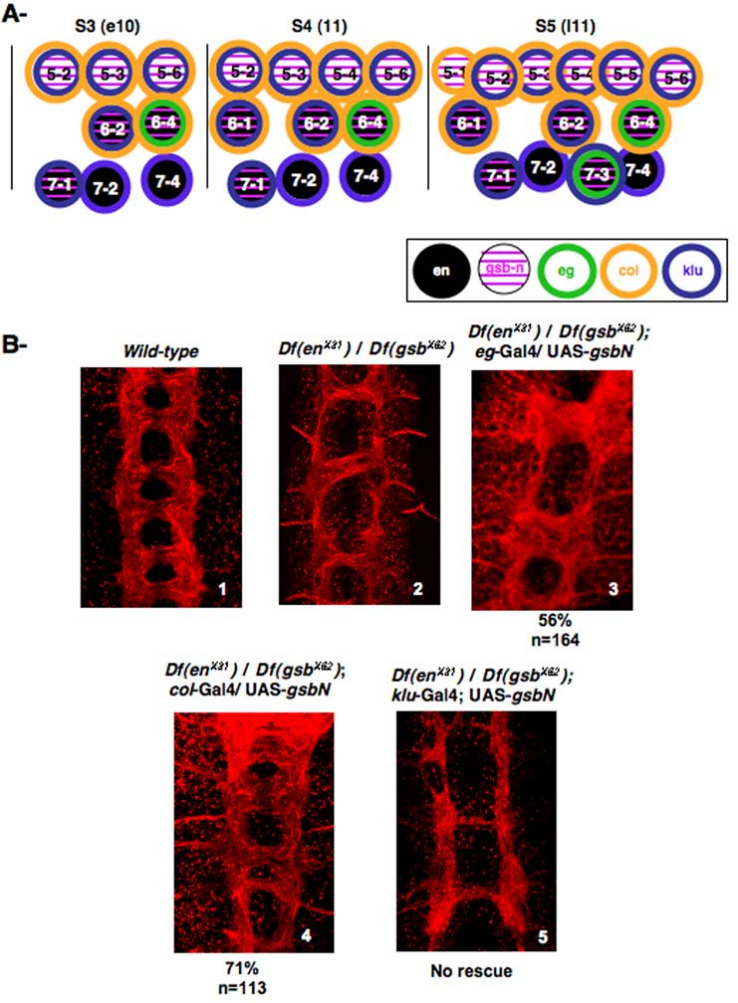
Identification of NBs involved in the formation of the PCs. A NB map of rows 5, 6 and 7, from S3 to S5 waves of delamination from early stage 10 (e10) to late stage 11 (l11), with indicated expression of EN (in black), GsbN (in pink), Eagle (in green), Collier (in yellow), and Klumpfuss (in blue). B Rescue experiments involving the reintroduction of GsbN into cells that are affected in the transheterozygous (*Df en^X31^/Df gsb^X62^*) embryos shown in (2), to compare to wild-type VNC shown in (1). Flat preparations are shown of stage 15 embryos labeled with a Cy3-conjugated anti-HRP antibody to visualize the VNC architecture (red). According to the diagram shown in A, GsbN expression was driven: (3) with *eagle*-Gal4 in NB 6-4 and NB 7-3; (4) with *collier*-Gal4 in several NBs, including NB 6-4, but not NB 7-3; and (5) with *Klumpfuss*-Gal4 in several NBs, including NB 7-3, but not NB 6-4.

According to the NB map (Figure 6A), which has been drawn according to Doe [5] and Broadus [25], and according to the results on Figure 4, EN and GsbN are expressed in NBs 6-1; 6-2; 6-4, and 7-1, and in 7-3 after the S5 wave of delamination. Since PCs are normally formed in (*Df en^X31^/+)* heterozygous embryos ([12] and data not shown), but not in (*Df en^X31^/Df gsb^X62^*) embryos (Figure 6B2), we asked which NBs required a normal level of GsbN expression to form the PCs.

To address this question, we used various Gal4 drivers to increase GsbN expression in specific NBs that were believed to be affected in (*Df en^X31^/Df gsb^X62^*) embryos. As shown in Figure 6A, we chose three Gal4 drivers that would restore GsbN expression in different NBs and analyzed the resulting VNC architectures.

The *eagle*-Gal4 driver was first used to restore GsbN expression in NBs 6-4 and 7-3 [32], in which both EN and GsbN are known to be expressed ([33] [16], and this study). Interestingly, we found that restoring GsbN expression in these NBs led to the formation of additional axons that crossed the midline through PCs (Figure 6B3) when compared to (*Df en^X31^/Df gsb^X62^*) embryos (Figure 6B2). Therefore, whereas the global shape of the embryos kept highly disturbed, the expression of GsbN in NBs 6-4 and 7-3 was sufficient to rescue both axon growth and crossing of the midline. In this case, 56% of the segments were rescued (n = 164), but all the embryos showed at least one segment presenting axon growth rescue. This suggested that the expression of EN and GsbN in NB 6-4 and/or NB 7-3 was necessary to trigger axon growth and midline crossing to further form the PC bundles.

In order to specifically determine whether NB 6-4 and/or NB 7-3 were responsible for this axon growth, we compensated for the GsbN defect using two other Gal4 drivers. One was a *collier*-Gal4 driver; *collier* is known to be expressed in a subset of NBs [34]. We first verified using double immunostainings with anti-EN and anti-Collier (provided by M. Crozatier) antibodies that Collier, even though broadly expressed, overlapped EN expression in row 6 but not in row 7 (data not shown). Also, immunostaining of *eagle*-Gal4/mCD8GFP embryos with the anti-Collier antibody confirmed that, among the *eagle*-positive NBs/neurons marked by membrane-associated GFP, Collier was expressed in NB 6-4 but not in NB 7-3 (data not shown). Therefore, using this Gal4 line, GsbN expression was restored in NB 6-4 but not in NB 7-3, as indicated in Figure 6A. With this driver, we found that 71% of the segments were rescued (n = 113) (Figure 6B4), indicating that GsbN expression in NB 6-4 is sufficient to rescue PCs that are missing in (*Df en^X31^/Df gsb^X62^*) embryos (Figure 6B2). However, we noted that GsbN expression in NB 6-4 was not able to completely reconstitute the process, since ACs and PCs were not always properly separated (Figure 6B3 and 6B4, compared to wild-type shown on Figure 6B1).

Finally, expression of GsbN was induced using a *klumpfuss*-Gal4 driver [35]. *klumpfuss* was previously shown to be expressed in most NBs, although not in NB 6-4 [36] (Figure 6A). Driving GsbN expression in these NBs, which include NB 7-3, was unable to rescue the loss of posterior PC seen in (*Df en^X31^/Df gsb^X62^*) embryos (n>100) (Figure 6B5).

To summarize, we were able to show that the missing PC phenotype could be rescued when GsbN expression was restored in both NB 6-4 and NB 7-3 (with the *eagle*-Gal4 driver), or in NB 6-4 alone (with the *collier*-Gal4 driver), indicating that expression in NB 6-4 is critical for PC formation. This was further confirmed using the *klumpfuss*-Gal4 driver, which restored GsbN in NB 7-3 but not in NB 6-4, and which was unable to rescue the formation of PCs.

Together, these experiments showed that co-expression of EN and GsbN in NB 6-4 can modify the cell's intrinsic abilities in ways that are necessary to form the PCs. We can notice that these expressions in NB 6-4 are not only involved in neuronal behavior of NB 6-4 progeny, but also in the behavior of neurons that normally project through PC, and that are also issued from other neuroblasts.

### Expression of GsbN is able to change axonal guidance

**Figure 7:**
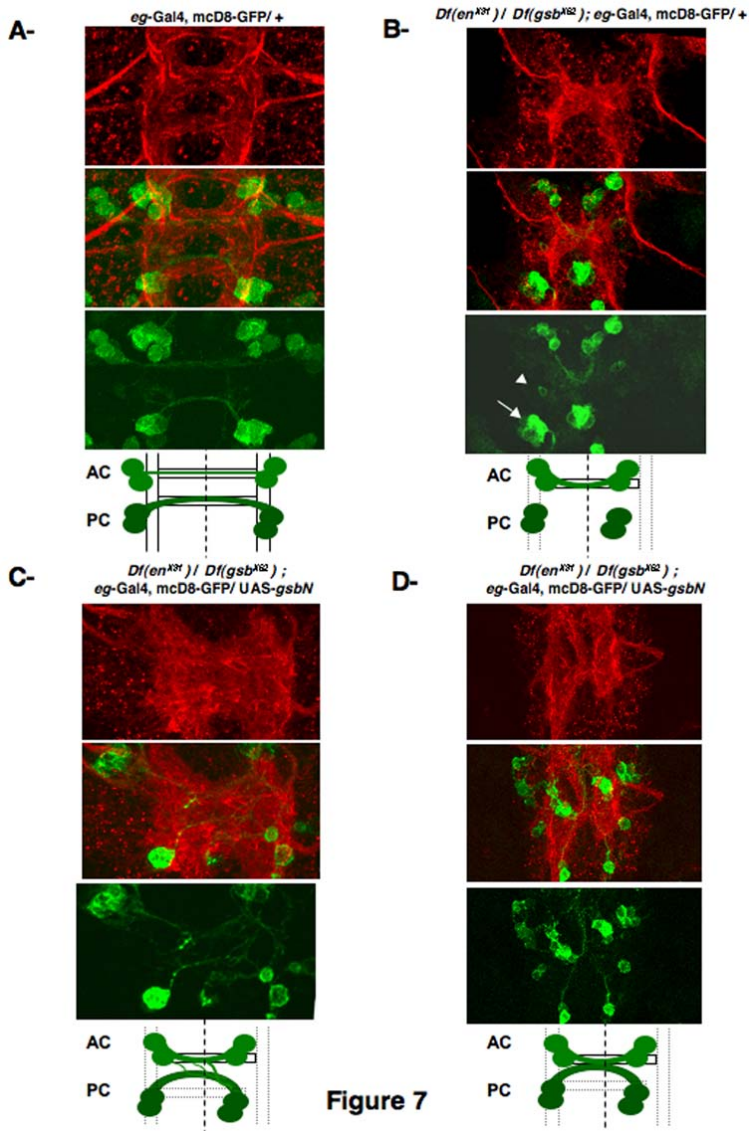
Behavior of *eagle*-positive neurons. Flat preparations are shown of stage 15 *eagle*-Gal4, UAS-mcD8-GFP embryos, labeled with a Cy3-conjugated anti-HRP antibody to visualize the VNC architecture (red) upper panels; and with a polyclonal anti-GFP antibody, secondarily detected by Cy2-anti rabbit (green) lower panels, with the merged images in the middle. To help the lecture of the phenotypes, a diagram is provided. Only one segment is shown, but the corresponding entire cords are provided on Figure S1. *eagle*-positive neuronal behavior is shown A in a wild-type background, B in the transheterozygous (*Df en^X31^/Df gsb^X62^*) background (arrowhead indicates neuronal progeny of the NB 6-4, and arrow indicates neuronal progeny of the NB 7-3), C and D in the transheterozygous (*Df en^X31^/Df gsb^X62^*) background, when restoring GsbN expression in *eagle*-positive cells. Note that when commissures appear thicker, in 56% of the segments, two types of results are obtained and correspond to the pictures provided in C and D.

We next analyzed more precisely the axonal behavior of a subset of neurons, in different genetic backgrounds. Following the axonal projection behavior of *eagle*-expressing neurons appeared to be the best system, since *eagle* is expressed in NB 6-4 and NB 7-3, whose progeny send their axons through PCs [37], as well as in NB 2-4 and NB 3-3, whose progeny send their axons through ACs [38]. The use of an *eagle*-Gal4 driver and a UAS-mCD8-GFP transgene, which encodes a membrane-associated GFP [39], allowed the visualization of axonal projections of the *eagle*-expressing neurons (Figure 7A). In transheterozygous (*Df en^X31^/Df gsb^X62^*) embryos, we confirmed, as shown on Figure 6B2, that only neuronal progeny projections through AC are formed (Figure 7B). Indeed NB 2-4 and NB 3-3 still project their axons and cross the midline, whereas axons issued from NB 6-4 and NB 7-3 neuronal progeny were not growing, even though *eg* positive cells are detected (Figure 7B. arrowhead indicates neuronal progeny of the NB 6-4, and arrow indicates neuronal progeny of the NB 7-3, see also Figure S1). This shows that loss of PC phenotype of transheterozygous (*Df en^X31^/Df gsb^X62^*) embryos, visualized by HRP staining, is associated with a lack of axonal growth. The rescue experiments of (*Df en^X31^/Df gsb^X62^*) embryos driven by *eagle*-Gal4/UAS-GsbN (shown in Figure 6B3) were reproduced in the presence of the UAS-mCD8-GFP transgene (Figure 7C, and Figure S1). When rescue occurs when visualized by anti-HRP immunostaining (in 56% of the segments, as mentioned on Figure 6B3), we can identify two types of images that are provided on Figure 7C and 7D. In both cases, when GsbN was expressed in these four *eagle*-expressing NBs (6-4; 7-3; 2-4; and 3-3), we were first able to confirm that axons from neurons that normally project through PCs (Figure 7C) grew properly and were projected towards the midline, as suggested by our previous results (Figure 6B3). However, we also detected that neuronal progeny from NB 2-4 and NB 3-3, when they ectopically express GsbN, were fasciculating with axons projecting through the PC (Figure 7C), leading sometimes to their fusion (Figure 7D). We also observed that this fusion was always occurring at the midline, making AC and PC trajectories unclear.

**Figure 8:**
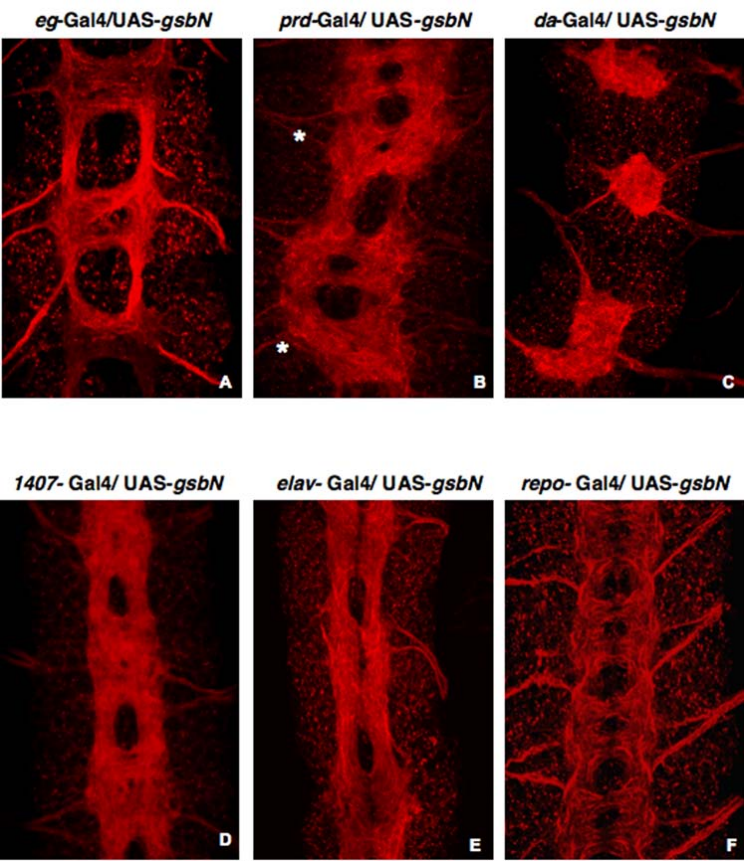
VNC architecture when GsbN is ectopically expressed. Flat preparations are shown of stage 15 embryos labeled with a Cy3-conjugated anti-HRP antibody to visualize the VNC architecture (red). GsbN expression is driven: A by *eagle*-gal4 driver, B in even segments (marked by *) by *paired*-Gal4, C early and ubiquitously by *daughterless-*Gal4; D in differentiated neurons by *1407-*Gal4, E by *elav*-Gal4, and F in glial cells by *repo*-Gal4.

This strongly suggested that GsbN expression in *eagle*-positive neurons, which normally project their axons through ACs, was sufficient to change their axonal pathfinding behavior, causing axons from ACs to fasciculate with PCs. Accordingly, ectopic expression of GsbN using the *eg*-Gal4 driver was sufficient to change the guidance of the axons and to lead to a fusion of the commissures (Figure 8A) that is reminiscent to the fusion phenotypes detected in (*Df en^X31^/Df gsb^X62^*; *eg*-Gal4/UAS-GsbN) rescue experiments (Figure 6B3 and Figure 7C). Driving GsbN in NBs, neurons, and glial cells using a *paired*-Gal4 driver led to a “fuzzy” commissure phenotype in even segments, in which the commissures were barely separated (Figure 8B). Further, when GsbN expression was driven in all cells prior to the formation of the VNC and the delamination of the NBs using a *daughterless*-Gal4 (Figure 8C) or *scabrous*-Gal4 driver (data not shown), axons were completely fused at the midline. Finally, the use of late drivers such as *1407*-Gal4 or *elav*-Gal4 (Figures 8D and 8E), which induce the expression of GsbN in already differentiated neurons, also resulted in fusion of the axons. Since fusion was thus detectable when GsbN was induced ectopically in differentiated neurons, it suggested that these axonal guidance problems involve a late function of GsbN. Since NB 6-4 gives rise to neuronal and glial progeny [38], we further tested if GsbN has also an influence through the glia. For this purpose, we analyzed the architecture of the VNC when driving GsbN expression in the glia (Figure S3). Ectopic expression of GsbN with a *repo*-Gal4 driver did not lead to any phenotype (Figure 8F), which excludes a role of GsbN in the glia, where GsbN is otherwise not expressed (Figure S2).

These results indicate that GsbN overexpression in neurons is sufficient to misroute axons to form axon bundles in the midline, and might correspond to a late function of GsbN that is independent of its early EN-related function in formation of PCs.

## Discussion

One of the most fascinating aspects of nervous system development is the establishment of stereotypic neuronal networks. An essential step in this process is the outgrowth and precise navigation of axons [1]. Most CNS growth cones initially head straight towards the midline, and only after crossing, they change their behavior as they turn and follow specific longitudinal pathways. In Drosophila, the majority of axons cross the midline within either anterior or posterior commissures. The formation of commissures starts at stage 12 of embryonic development and involves dynamic, but reproducible interactions between: growth of the neurons, their fasciculation with other neurons to form the different bundles, apoptosis of neuronal cells, and migration of glial cells. In Drosophila, formation of posterior and anterior commissures are not believed to be related, and different cells and possibly different signals appear to be used for the guidance of the different commissures [40] [41]. Each neuron makes a choice as whether to cross the midline and, for those that do cross, whether to grow through the anterior or the posterior commissure, where axons are arranged in fascicles. One central issue is the identification of the intrinsic pathfinding abilities at the different steps of the neural development that are involved in the differential neuronal behavior. Whereas the process of construction of longitudinal tracts has been previously analyzed [42], [43], [44], as has the formation of ACs [45], [46], little is known about the formation of the PCs.

Obvious candidates for organizing the intrasegmental distribution of guidance cues along the antero-posterior axis are the segment polarity genes. Consistent with this assumption, embryos mutant for EN/INV and for Gsb/GsbN have severely reduced, and often missing, posterior commissures ([8] [12], and this study). Segment polarity genes occupy an intriguing position within the segmentation hierarchy. They are required in the epidermis to specify cell fates within each segment, and are also active both before and during the delamination of neuroblasts to generate the CNS. In particular, the specification of neuroblast identity within a given hemisegment depends upon interactions between segment polarity genes such as Engrailed and Invected with Gsb [9] [37] [16]. Whereas *gsb* is expressed at early stage 6 and begins to be detectable when NBs start to delaminate (stage 9), GsbN is only detectable starting in stage 10 embryos and appears simultaneously to the disappearance of Gsb [17]. EN and GsbN are expressed in NBs of rows 6 and 7. Interestingly, NB 6-4 appears during the S3 wave of delamination at stage 10, just as GsbN expression begins. The axons that pioneer the first tracts will appear later, at stage 12, by which time Gsb expression is nearly completely switched off [17].

In this report, we first have developed several lines of evidence for a concerted action of EN and GsbN in neuroblasts. Indeed, we have been able to show that whereas heterozygous *en/inv* or *gsb/gsbN* deletions (respectively (*Df en^X31^/+*) and (*Df gsb^X62^ /+))* show a normal architecture of the VNC, double heterozygotes (*Df en^X31^/Df gsb^X62^*) do not form PCs properly, resulting with high penetrance in loss of PCs. This result clearly indicates that EN/INV and Gsb/GsbN act together to form PCs. EN has already been shown to have a major function in PC formation, comparatively to INV [12]. In view of our observation of physical interactions between EN protein and GsbN protein, we analyzed whether GsbN might be responsible for the absence of PCs in the transheterozygous (*Df en^X31^/Df gsb^X62^*) genetic background. Using rescue experiments, we indeed found that expression of GsbN was able to rescue the phenotype. This shows that, genetically, EN and GsbN act together to build the posterior PC commissures, which are part of the VNC. We have several reasons to suspect that GsbN might act as a cofactor of EN for PC formation. First, we have not found any evidence for a direct regulation of EN on *gsbN*, since no EN binding fragments were isolated within the GsbN locus by chromatin immunoprecipitation [21]. This corroborates our observation that EN does not bind the GsbN locus (60F region) on polytene chromosomes (Figure 3 and [19]). Moreover, we have been able to show that missing PC phenotype resulting from EN misexpression is not associated with a loss of *gsbN* function (data not shown). In addition, as shown in this report, EN and GsbN proteins interact *in vitro* (as evidenced by GST-pull down and coIP experiments), in yeast (demonstrated using a two-hybrid assay), and *in vivo* in Drosophila, since they were found to bind common loci on polytene chromosomes. Together, these results support the notion that EN and GsbN act as cofactors in the construction of the VNC. Interestingly, we were only able to rescue the missing PC phenotype of transheterozygous (*Df en^X31^/Df gsb^X62^*) embryos when GsbN was restored from early stages in neuroblasts, but not in differentiated neurons. This shows that the formation of the PCs involves an early function of GsbN, which is consistent with a concerted action with EN, since we previously showed that the early function of EN is responsible for PC axon growth [12]. We know from previous studies that PCs are formed from neurons originating in rows 6 and 7 (which express both EN and GsbN), as well as from neurons issued in other rows, such as row 5 (that only express GsbN) [26], [27]. These observations strongly suggest that NBs expressing both the EN and GsbN transcription factors might contain instructions for PC formation. In a first step, EN/INV and Gsb (not GsbN) were shown to be involved in NB specification [9] [37]. In particular expressions of EN and Gsb were found to be necessary in the formation of NB 6-4 [16]. However, since *gsbN* is not expressed in the ventral neuroectoderm during the time of NB specification, it hence cannot play a role in neural specification at this level [17], [8]. Therefore, we expect the interaction between EN and GsbN not to interfere directly in the formation and segregation of the NBs, but rather to happen after the NBs are formed. In particular, we show here that EN and GsbN are involved in the further determination of NB 6-4 to form posterior commissure. Indeed, as shown in this report, EN and GsbN functions in NB 6-4 not only influence NB 6-4 behavior, but also the behaviors of other neurons that construct the PCs, strongly suggesting that this concerted action of EN and GsbN is involved in triggering formation of the PC bundles. One hypothesis is that they act together in a same complex to activate functions that are required for the development of the NBs and that will be necessary for further axon growth and pathfinding. Indeed, driving GsbN in NB 6-4 using different drivers such as *eagle*-Gal4 or *collier*-Gal4 was sufficient to rescue axonal growth and crossing of the midline of the PC formers in the transheterozygous (*Df en^X31^/Df gsb^X62^*) background.

However, whereas axon growth and crossing of the midline seem to be rescued in both cases, separation between ACs and PCs were incomplete. One possible explanation for the fusion of the commissures, was provided by our analysis of the neuronal behavior of *eagle*-positive neurons. When GsbN is expressed in *eagle*-expressing NBs/neurons (corresponding to NB 6-4 and NB 7-3 progeny projecting through PCs, and to NB 2-4 and NB 3-3 progeny projecting through ACs), we not only observed a rescue of axonal growth of PCs, but also found that neurons projecting through ACs were fasciculating with the PCs. This suggests that the “fuzzy” separation of ACs and PCs observed with the *eagle-*Gal4 driver probably resulted from abnormal axonal pathfinding in ACs. Therefore, formation of PC bundles requires at later stages, a specific function of GsbN in the neurons that is driving the fasciculation and guidance of axons forming PC commissures. This latter function of GsbN might correspond to a late function, since expression of GsbN with both early acting (in NBs, neurons, and glial cells) and late acting (in differentiated neuronal cells) Gal4 drivers was found to misroute axons that would have otherwise fasciculated to other axons at the midline. In this case too, all the axons seem to fasciculate, leading to a fuzzy separation of the commissures, sometimes collapsing at the midline. These observations support the idea that this late function of GsbN in the neurons is involved in their axonal pathfinding and in formation of fasciculations that are required to form the bundles, and that are a property of the follower neurons. EN function on axonal pathfinding was found to occur early in the neuroblasts, but not in differentiated neurons [12]. Expression of GsbN in differentiated neurons was also not able to trigger axonal growth and crossing of the midline of PC formers (Figure 5B3). Therefore, we can conclude for a two-step involvement of GsbN in the formation of the PCs. At first, a concerted action of EN and GsbN is necessary in NB 6-4 to trigger the axon growth of PC formers, whereas axonal guidance *per se* might rather result from independent role of EN and GsbN, a specific action of GsbN on guidance occurring in differentiated neurons.

Important questions relate to the behavior of NB 6-4 in different genetic contexts and the exact role of EN and GsbN in this process. Since NB 6-4 generates both neurons and glial cells [28], one hypothesis is that they act together in the glial cells that are known to play a crucial role in axonal guidance [45] [47] [44]. Several hypothesis could be drawn: i) GsbN expression is needed to form NB 6-4 progeny. However, in transheterozygous (*Df en^X31^/Df gsb^X62^*) embryos, *eg* expressing neuronal cells were formed, but their axons were not growing (Figure 7B). As well, we found that glial cells issued from NB 6-4 were formed (Figure S4), which does not favor this hypothesis. ii) GsbN acts directly on glial cell function. However whereas EN is expressed in the glia [16] [48], we found that GsbN was not expressed in the glia (Figure S2), which also excludes this hypothesis. iii) EN and GsbN are activating a function that will be expressed in NB 6-4 glial progeny and that is triggering axon growth and crossing of the midline, making these particular glial cells central in this process. However ectopic expression of GsbN in all the glia does not lead to abnormal architecture of the VNC (Figure 8F, and Figure S3), which does not favor for an indirect effect of GsbN in the glia. iv) Finally, functions activated by EN and GsbN in NB 6-4 will be used in its neuronal progeny to “show the way” of GsbN expressing neurons. Our data rather favor for a central role of NB 6-4 neuronal progeny to trigger the formation of the PCs. This of course does not exclude, as shown for longitudinal tracts [44], for a crucial role between on one hand these particular neurons and the NB 6-4 glial progeny, followed by a crosstalk between these glial cells and the GsbN expressing neurons.

The molecular mechanisms involved in these processes will be particularly informative in our understanding of how neuronal axon trajectories are dictated to construct the VNC. The next challenge will be also to understand what cellular events and downstream functions are regulated in NBs by both EN and GsbN to construct PC bundles, since their expression in NB 6-4 seems to be crucial to trigger the whole process of PC formation, and what are the specific downstream functions regulated more specifically by GsbN to specify fasciculations between GsbN expressing neurons, a property associated to the followers.

One way to address these questions would be to identify genes that are directly regulated by EN and GsbN and that would therefore likely be misregulated in the transheterozygous (*Df en^X31^/Df gsb^X62^*) genetic background.

Finally, the identification of direct targets of GsbN or of common direct targets of EN and GsbN would allow a better understanding of the downstream functions involved in the specification and differentiation of the different neurons, which ultimately drive axon growth and axonal pathfinding.

The observations that vertebrate homologs of EN (*EN1* and *EN2*) and Gsb/GsbN (*Pax3* and *Pax7*), but also other *Pax* genes are required in neural fate specification [49], that they are expressed in the same cells [50], and that they are involved in axon growth [51], strongly suggests that the molecular mechanisms acting in Drosophila are relevant to and probably conserved in higher organisms.

## Materials and Methods

### *Drosophila* strains

Crosses were usually raised at 25°C, except rescue experiments which were analyzed at both 25°C and 21°C.

The Hs-GsbN strain was obtained from Markus Noll [52]. *Df(2R)IIX62 (gsb^X62^*) [17] corresponds to a deficiency which removes both *gsb* genes and was obtained from Bloomington Stock Center, as was the UAS-mcd8-GFP line on chromosome III [39]. The Hs-EN strain [53], UAS-EN strain [54], and *Df(2R)SFX31 (en^X31^)* [55] were provided by Thomas Kornberg. Mutations were balanced with a chromosome marked with *krüppel*-GFP [56] in order to select for transheterozygous (*Df en^X31^/Df gsb^X62^*) embryos. Note that around 30% of transheterozygous (*Df en^X31^/Df gsb^X62^*) embryos do not show any phenotype. Therefore penetrance of the phenotypes and of the rescue was calculated on affected embryos, easily recognizable because rescue did not concern all the segments.

The UAS-*gsbN* lines were constructed. *gsbN* cDNA was isolated by PCR performed on the RE64348 clone (from the DGC gold collection) using the following primers: GGGGTACCCATTCGGGACCAT and TGCTCTAGAAATCATGACCA, and was cloned into the pUAST vector. pUAST-*gsbN* was sequenced and transgenic lines obtained after injection into *w^1118^* embryos.

The following Drosophila Gal4 lines were used: MS1096-Gal4 [57]; *paired*-Gal4 [29]; *daughterless*-Gal4 [30]; 1407-Gal4 [31]; *eagle*-Gal4 [32]; *Collier*-Gal4, which corresponds to a transgenic line in which Gal4 is under the control of 10 kb of the Collier promoter region (P10-Gal4), was provided by Michele Crozatier [58]; *Klumpfuss*-Gal4 (*klu^G410^*) was provided by Thomas Klein [35].

### Two-Hybrid screen

An embryonic *Drosophila melanogaster* Matchmaker cDNA library (Clontech) was used for the screen, in which cDNA inserts are cloned in-frame with the GAL4 activation (AD) domain in the pACT2 vector. The MAT*α*Y187 yeast strain was transformed with 50 µg of the library and plated on leucine-deficient plates. The pGBT9-EN bait construct produced Engrailed protein in frame with the Gal4 DNA binding domain (DB). The yeast two hybrid screen was performed, using the mating procedure [59], with minor modifications. For the screen, thawed cells from the library (corresponding to 5×10^7^ independent clones) were mixed with MAT*a* strain cells (CG1945) transformed with the Engrailed bait plasmid, plated on complete medium for 5 h, and transferred onto Tryptophan/Leucine/Histidine deficient plates (DO-W-L-H) supplemented with 2.5 mM 3′-amino triazol (3AT). After 3 to 5 days, a β- Galactosidase overlay test was performed on the Histidine-positive clones. Blue positive clones were then streaked onto DO-W-L-H/3AT plates. PCR amplification of the inserts using AD specific primers was performed on the yeast colonies, and PCR products were sequenced. ß-Galactosidase and Histidine tests were further performed using full-length Engrailed protein (EN-FL) synthesized in frame with the DB (pGBT9), with either full-length GsbN (GsbN-FL, corresponding to 449 aa), or with the C-terminal region of GsbN (GsbN-Cter, encompassing aa 173 to aa 449, which contains the homeodomain region located from aa 184 to aa 241, but otherwise does not share homologies with Gsb) [60], cloned in the Gal4 AD (pGAD vector).

### GST pull-down and co-immunoprecipitation assays

GST fusion proteins were produced according to manufacturer's instructions (Pharmacia). The TNT expression system (Promega) was used to produce S^35^-labelled GsbN (GsbN*) or EN (EN*) full-length proteins.

For GST pull-down assays, GST, GST-GsbN and GST-EN proteins, immobilized on glutathione agarose beads (Sigma), were incubated for 30 min at room temperature in TBST (140mM NaCl, 20mM Tris pH 7.6, 2mM EDTA, 0.1% Triton X-100, 25mM Glycerol Phosphate, 10% glycerol, 2mM NaPPi) containing 2% BSA. Beads were incubated in the presence of appropriate S^35^-labelled proteins for 1hr at room temperature in TBST containing 0.5% Triton X-100 and 0.2% BSA. Washes (4×10 min) were done in TBST containing 1% Triton X-100 and increasing concentrations of NaCl (250, 500, 750 mM and 1M). Beads were collected by centrifugation at 2000 rpm for 2 min between each wash. Beads were resuspended in Laemmli buffer, boiled, and loaded on an SDS polyacrylamide gel. Gels were treated for 30 min in 1M salicylic acid containing 2.5% glycerol, dried and autoradiographed.

### Antibodies and Immunostainings

The anti-EN antibodies used in this study correspond either to the mouse monoclonal 4F11 provided by Nipam Patel [24], which was used on embryos at a 1∶50 dilution, or to a rabbit polyclonal anti-EN antibody, which was raised against a truncated form of EN lacking the homeodomain and used on chromosome squashes at a dilution of 1∶40.

Anti-GsbN corresponded to polyclonal antibodies. One was made in guinea pigs, against the entire protein, and was used on chromosome squashes at a dilution of 1∶10 and in embryos at a dilution of 1∶400. A specific anti-GsbN was also prepared in rabbits, against two specific peptides (NH2-CYSHPLPTQGQAKYWS-COOH and NH2-CRGSDRGSEDGRKDYT-CONH2) that are present in a region that does not share homology with the Gsb protein. The specificity of the antibody and the absence of cross-reactivity with Gsb was verified by western blot (data not shown). This antibody was used for xon aimmunoprecipitation at a dilution of 1∶1000.

Polyclonal rabbit anti-GFP (from Molecular Probes) was used at a dilution of 1∶1000, and goat Cy3-conjugated anti-HRP (from Jackson ImmunoResearch) was used at a 1∶100 dilution.

Secondary antibodies were: Cy3 anti-Rabbit, Cy2 anti-Mouse, Cy2 anti-Guinea-pig, and Cy2-anti-Rabbit (from Jackson ImmunoResearch), and were used at a dilution of 1∶1000.

Preparations of flat dissected preparations of embryonic Ventral Nerve Cord: Slides are coated with 0.1% Polylysine (Sigma). On each slide, silicon cement forms a bath. Staged dechorionated embryos from stage 15 to stage 17 are dried before being deposited onto double-sided tape placed on the polylysine slide. Embryos are arranged in the same orientation, with the ventral face on the tape. Using a tungsten needle, embryos are opened from A to P and placed and spread onto the polylysine coat, in order to remove the internal organs. Embryos are then fixed in PBS+4% PFA (Electron Microscopy Sciences) for 20 to 40 min. Washes (2×20 min) are performed in PBS+Triton 0.1%, avoiding drying the embryos. Blocking is performed in PBS+BSA 1%, before immunostainings which were performed as described in Joly at al. (2007) [12]. All washes and incubations in the bath were performed in 400 µl.

Squashes of polytene chromosomes from L3 larval salivary glands and immunostainings were performed according to Serrano et al. (1995) [18].

## Supporting Information

Figure S1 Behavior of eagle-positive neurons. Flat preparations are shown of stage 15 eagle-Gal4, UAS-mcD8-GFP embryos, labeled with a Cy3-conjugated anti-HRP antibody to visualize the VNC architecture (red); and with a polyclonal anti-GFP antibody, secondarily detected by Cy2-anti rabbit (green), with the merged images. eagle-positive neuronal behavior is shown. 1-3 in transheterozygous (Df enX31/Df gsbX62) embryos. 4-9 in transheterozygous (Df enX31/Df gsbX62) background, in the presence of GsbN in eagle-positive cells, corresponding to different images obtained in a context of the rescue. Brackets indicate the segments shown on Figure 7.
(3.73 MB TIF)

Figure S2 Exclusive expression of GsbN and Repo. Flat preparations are shown of stage 15 wild-type embryos. Embryos are labeled with anti-GsbN (in green) and anti-Repo (in red). 1–3 and 4–6 show two different confocal planes, where we can detect in 3 and 7 merged images that NB 6-4 lateral glial cells progeny do not express GsbN (arrows), as well as NB 6-4 medial glial cells progeny (arrowheads). This confirms that GsbN is not expressed in the glia, and more specifically not in NB 6-4 glial cells progeny.
(1.78 MB TIF)

Figure S3 Normal VNC architecture when GsbN is ectopically expressed in the glia. Flat preparations are shown of stage 15 repo-Gal4, UAS-GsbN embryos, labeled with 1- anti-Repo (in red), 2- anti-GsbN (in green), 3- corresponds to the merged images of 1 and 2, showing that most of the glia express GsbN, 4- anti-HRP (in pink), showing that this ectopic expression of GsbN in the glia did not affect the architecture of the VNC.
(1.94 MB TIF)

Figure S4 Analysis of Repo expression in different genetic backgrounds. Flat preparations are shown of stage 15 eagle-Gal4, UAS-mcD8-GFP embryos, labeled with anti-Repo (in red) and anti-GFP (in green). 1–3 in eg-Gal4, UAS-mcD8-GFP. 4–6 in Df enX31/Df gsbX62; eg-Gal4, UAS-mcD8-GFP. 7–9 in Df enX31/Df gsbX62; eg-Gal4, UAS-mcD8-GFP/UAS-GsbN. On merged images, we identified eg positive cells that correspond to glial cells (arrows). This confirms that NB 6-4 glial cells are formed in transheterozygous (Df enX31/Df gsbX62) embryos (arrows in 6) and in rescue context (arrows in 9).
(3.32 MB TIF)
