# Supplementary figures and images for: A Concerted Action of Engrailed and Gooseberry-Neuro in Neuroblast 6-4 Is Triggering the Formation of Embryonic Posterior Commissure Bundles
Source: PLoS One. 2008 May 21;3(5):e2197. doi: 10.1371/journal.pone.0002197 (PMC2373891; doi:10.1371/journal.pone.0002197)

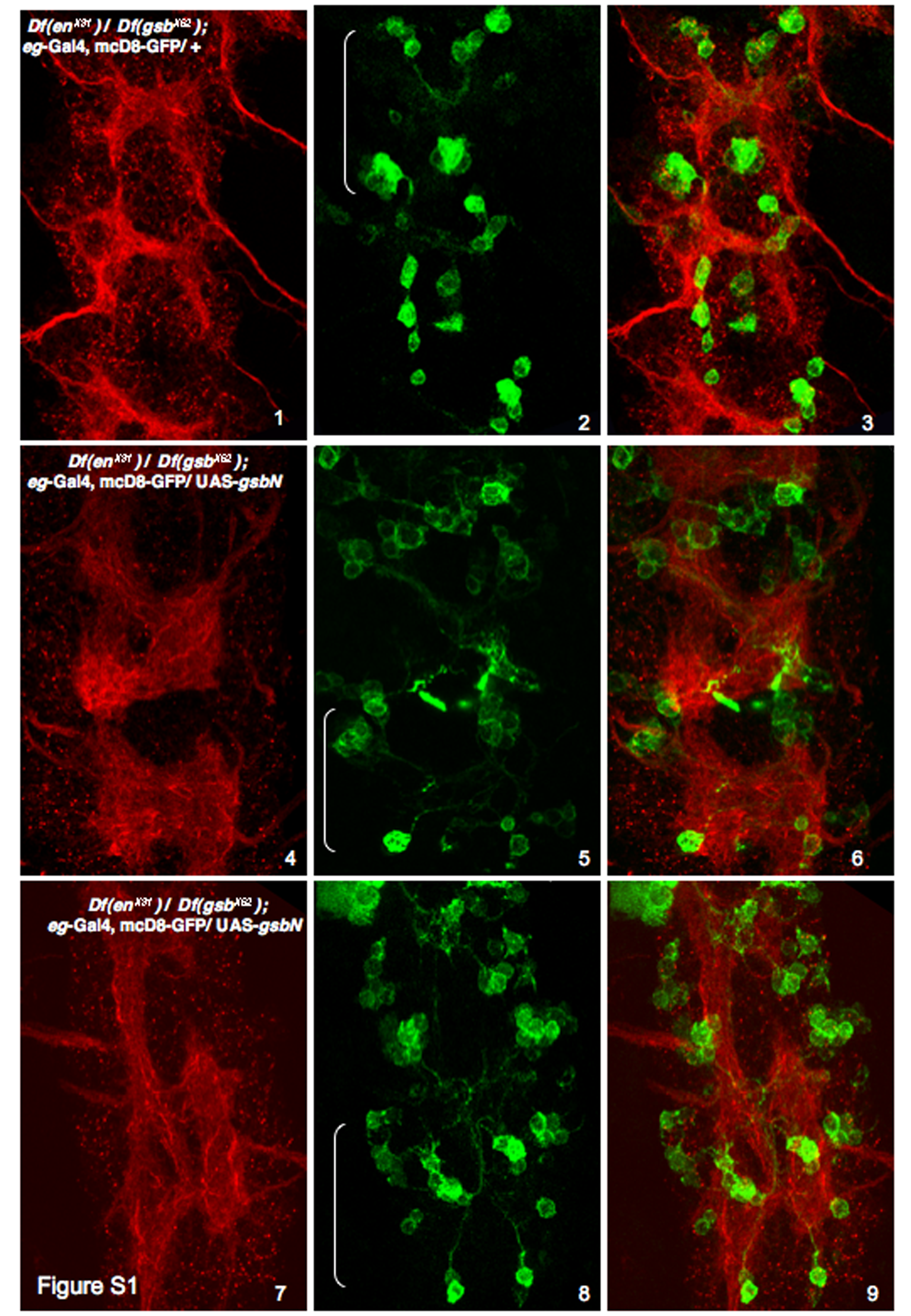

Supplement: Figure S1 — Behavior of eagle-positive neurons. Flat preparations are shown of stage 15 eagle-Gal4, UAS-mcD8-GFP embryos, labeled with a Cy3-conjugated anti-HRP antibody to visualize the VNC architecture (red); and with a polyclonal anti-GFP antibody, secondarily detected by Cy2-anti rabbit (green), with the merged images. eagle-positive neuronal behavior is shown. 1-3 in transheterozygous (Df enX31/Df gsbX62) embryos. 4-9 in transheterozygous (Df enX31/Df gsbX62) background, in the presence of GsbN in eagle-positive cells, corresponding to different images obtained in a context of the rescue. Brackets indicate the segments shown on Figure 7. (3.73 MB TIF) [file pone.0002197.s001.tif]

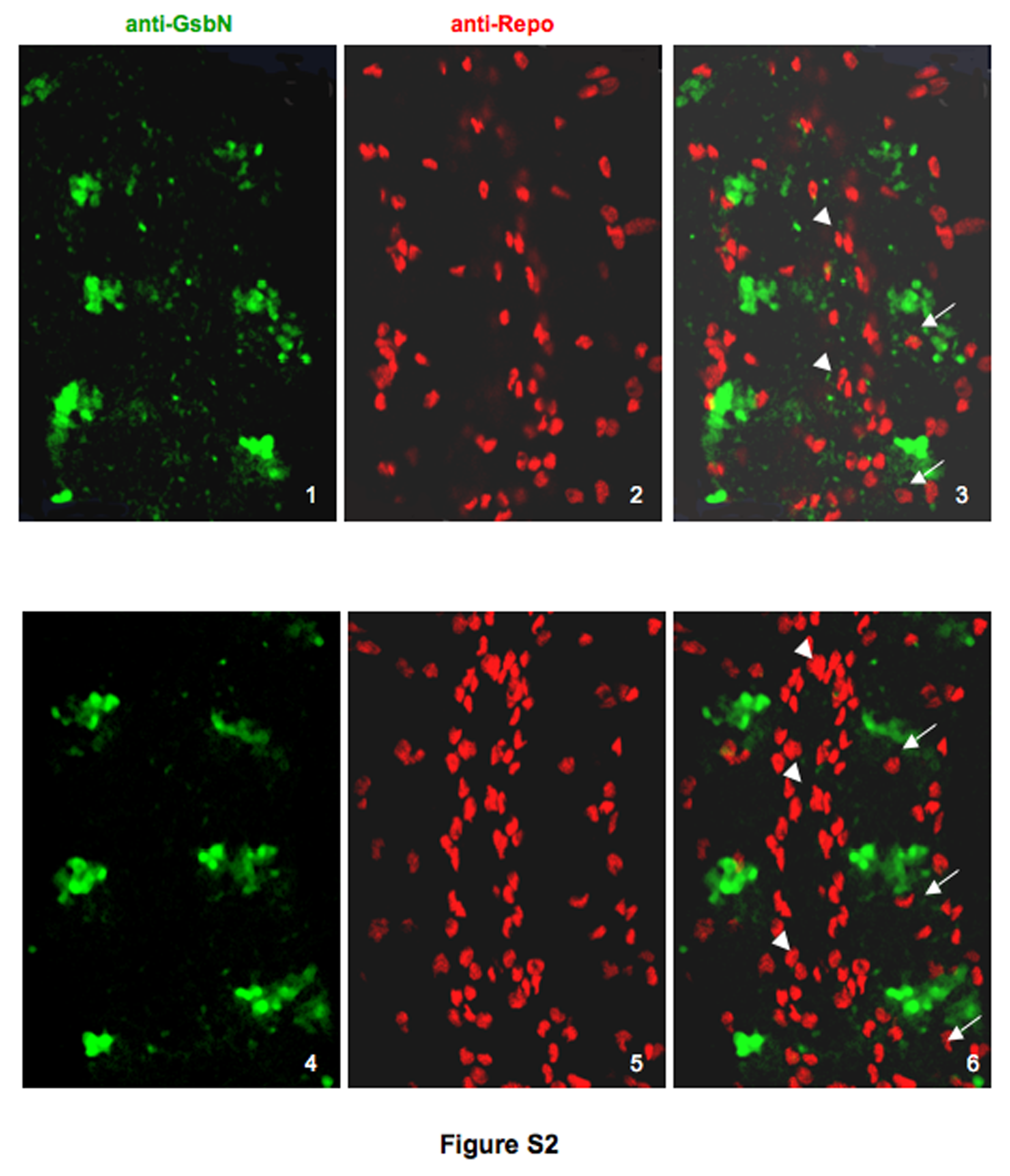

Supplement: Figure S2 — Exclusive expression of GsbN and Repo. Flat preparations are shown of stage 15 wild-type embryos. Embryos are labeled with anti-GsbN (in green) and anti-Repo (in red). 1–3 and 4–6 show two different confocal planes, where we can detect in 3 and 7 merged images that NB 6-4 lateral glial cells progeny do not express GsbN (arrows), as well as NB 6-4 medial glial cells progeny (arrowheads). This confirms that GsbN is not expressed in the glia, and more specifically not in NB 6-4 glial cells progeny. (1.78 MB TIF) [file pone.0002197.s002.tif]

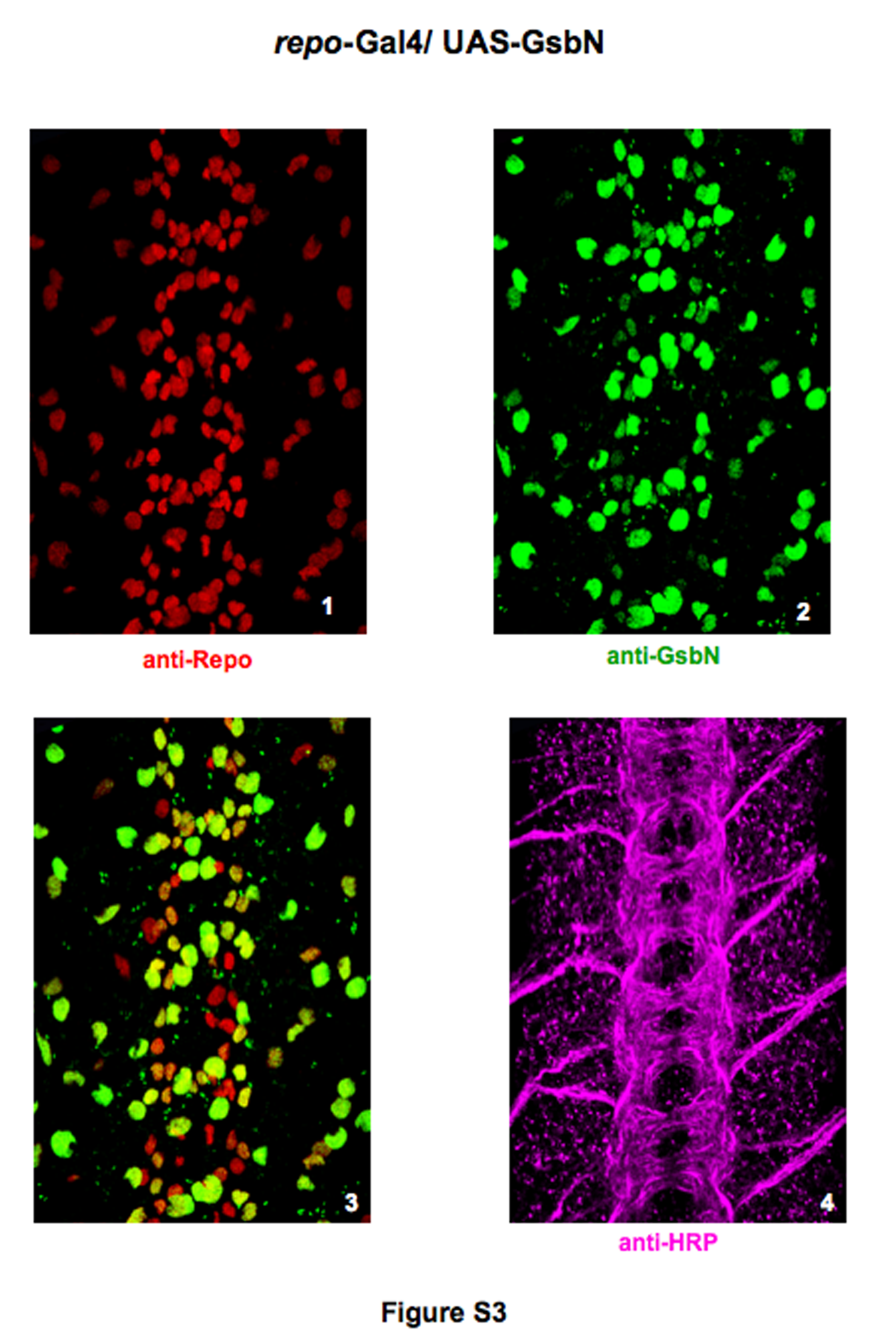

Supplement: Figure S3 — Normal VNC architecture when GsbN is ectopically expressed in the glia. Flat preparations are shown of stage 15 repo-Gal4, UAS-GsbN embryos, labeled with 1- anti-Repo (in red), 2- anti-GsbN (in green), 3- corresponds to the merged images of 1 and 2, showing that most of the glia express GsbN, 4- anti-HRP (in pink), showing that this ectopic expression of GsbN in the glia did not affect the architecture of the VNC. (1.94 MB TIF) [file pone.0002197.s003.tif]

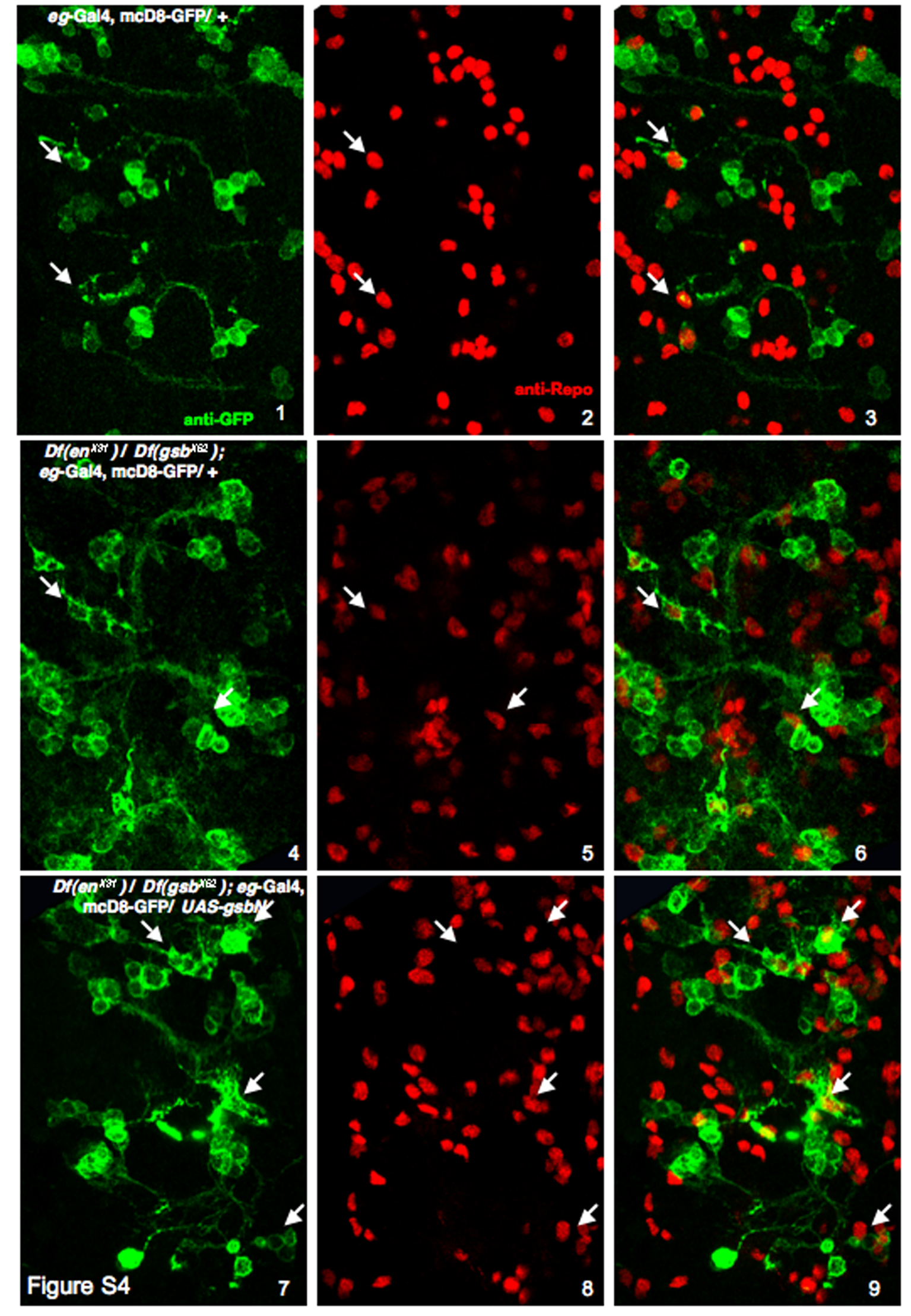

Supplement: Figure S4 — Analysis of Repo expression in different genetic backgrounds. Flat preparations are shown of stage 15 eagle-Gal4, UAS-mcD8-GFP embryos, labeled with anti-Repo (in red) and anti-GFP (in green). 1–3 in eg-Gal4, UAS-mcD8-GFP. 4–6 in Df enX31/Df gsbX62; eg-Gal4, UAS-mcD8-GFP. 7–9 in Df enX31/Df gsbX62; eg-Gal4, UAS-mcD8-GFP/UAS-GsbN. On merged images, we identified eg positive cells that correspond to glial cells (arrows). This confirms that NB 6-4 glial cells are formed in transheterozygous (Df enX31/Df gsbX62) embryos (arrows in 6) and in rescue context (arrows in 9). (3.32 MB TIF) [file pone.0002197.s004.tif]
